# Distinctive Regulatory T Cells and Altered Cytokine Profile Locally in the Airways of Young Smokers with Normal Lung Function

**DOI:** 10.1371/journal.pone.0164751

**Published:** 2016-10-31

**Authors:** Mahyar Ostadkarampour, Malin Müller, Johan Öckinger, Susanna Kullberg, Anders Lindén, Anders Eklund, Johan Grunewald, Jan Wahlström

**Affiliations:** 1 Respiratory Medicine Unit, Department of Medicine Solna, Karolinska Institutet, Stockholm, Sweden; 2 Rheumatology Research Unit, Department of Medicine Solna, Karolinska Institutet, Stockholm, Sweden; 3 Center for Molecular Medicine (CMM), Karolinska Institutet, Stockholm, Sweden; 4 Unit for Lung and Airway Research, Institute of Environmental Medicine (IMM), Karolinska Institutet, Stockholm, Sweden; 5 Karolinska University Hospital, Stockholm, Sweden; University of Rochester Medical Center, UNITED STATES

## Abstract

Smoking influences the immune system in different ways and, hypothetically, effects on pulmonary effector and regulatory T cells emerge as potentially detrimental. Therefore, we characterized the frequencies and characteristics of CD4+ and CD8+ T cell subsets in the blood and lungs of young tobacco smokers. Bronchoalveolar lavage (BAL) and peripheral blood were obtained from healthy moderate smokers (n = 18; 2–24 pack-years) and never-smokers (n = 15), all with normal lung function. Cells were stimulated *ex vivo* and key intracellular cytokines (IFNγ, IL-17, IL-10 and TNFα) and transcription factors (Foxp3, T-bet and Helios) were analyzed using flow cytometry. Our results indicate that smoking is associated with a decline in lung IL-17+ CD4+ T cells, increased IFNγ+ CD8+ T cells and these alterations relate to the history of daily cigarette consumption. There is an increased fraction of Foxp3+ regulatory T cells being Helios- in the lungs of smokers. Cytokine production is mainly confined to the Helios- T cells, both in regulatory and effector subsets. Moreover, we detected a decline of Helios+Foxp3- postulated regulatory CD8+ T cells in smokers. These alterations in the immune system are likely to increase risk for infection and may have implications for autoimmune processes initiated in the lungs among tobacco smokers.

## Introduction

The lungs are a unique immunologic organ which is constantly exposed to microorganisms and environmental irritants that trigger immune mechanisms. Inefficient or exaggerated immune responses to these environmental irritants may result in pathological conditions. The causative involvement of tobacco smoking in the pathogenesis of several diseases such as lung cancer, cardiovascular diseases, chronic obstructive pulmonary disease (COPD) and autoimmune disorders is widely recognized [1]. However, the fact that tobacco smoke includes thousands of compounds with the potential to influence the immune system is likely to drive mechanistic research for years to come.

The inhalation of tobacco smoke components influences the function of both structural and immune cells in the lungs, exerting both stimulatory and inhibitory actions. This leads to the recruitment of immune cells and inflammation but also to aberrations in the immune system, resulting in reduced immunity to infections [2, 3].

In the lungs, T helper lymphocytes, and in particular Th17 cells, emerge as pivotal in orchestrating host defense and acute inflammation following triggering by specific antigens, in certain cases leading to chronic inflammation and autoimmunity [4]. Interestingly, smoking is associated with both stimulation and inhibition of mediators that influence the distribution of T cell subsets in the lungs [1, 5]. Based upon the immunoreactivity for IL-17 in airway cells, tobacco smoking promotes IL-17 producing cells locally [6] and hypothetically this may contribute to the local accumulation of innate effector cells [6, 7].

To balance the activity of Th17 and other effector T cells, transcription factor Forkhead box protein 3 (Foxp3) positive regulatory T cells (Tregs) exert a suppressive function, thereby maintaining the homeostasis of the immune system [8].

Lineage-specific transcription factors are known to play a crucial role for the gene expression and function of T cells subsets, such as Th1, Th2, Th17 and Tregs. Helios is a member of the Ikaros transcription factor family and it has been considered as a marker of natural or thymus-derived Treg cells [9]. However, this has been challenged [10] and Helios has been suggested to be a marker of T cell activation and proliferation or even anergic effector cells [11, 12].

Tentatively, even though it is clear that pulmonary effector and regulatory T cells are affected in smokers with COPD, it remains unclear to what extent smoking *per se* may account these immune alterations in young subjects with normal lung function. To address this, we characterized the frequencies and characteristics of CD4+ and CD8+ T cell subsets in the blood and lungs of clinically healthy young tobacco smokers and never-smokers with normal lung function.

## Materials and Methods

### Study subjects and characterization

The study was approved by the Regional Ethical Review Board (Stockholm, Sweden). Oral and written informed consent was obtained from all subjects. A total of 18 smokers and 15 never-smokers of relatively young age were recruited via the Respiratory Medicine Unit (Karolinska University Hospital, Solna, Stockholm, Sweden). All subjects were examined by physician. Upon clinical examination they had no signs of infection and denied having any infection the last four weeks preceding the investigation. Nothing abnormal was seen on inspection of mouth and throat. Heart- and lung status examined by a stethoscope were normal. Laboratory tests including white and red blood cell counts, electrolytes, creatinine and C-reactive protein (CRP) were normal. They all had a normal chest X-ray and a normal lung function measured by dynamic spirometry (Jaeger MasterScope, Intramedic; Medikro PRO, Aiolos). We collected peripheral blood (PB) and bronchoalveolar lavage (BAL) samples obtained during bronchoscopy, as described previously [13]. The basic clinical, physiological and BAL cell characteristics are presented in Table 1.

**Table 1:** Features and BALF characteristics of the subjects.

	Never-smokers	Smokers
**Subject Characteristics**		
Number of individuals	15	18
Sex (Male/Female)	7/8	5/13
Age (Year)	24 (20–34)	26.5 (19–50)
Historic smoking (cigarettes/day)	-	10 (5–30)
Pack-years	-	5 (1.8–24.5)
**Pulmunary Function Tests**		
FVC (% of predicted)	106 (91–120)	107.5 (84–127)
FEV1 (% of predicted)	104 (82–120)	103 (80–113)
FEV1/FVC	0.82 (0.78–0.89)	0.80 (0.75–0.88)*
**BAL Specificities**		
BAL recovery%	78 (44–83)	68.5 (25–76)**
Cell concentration (million/Liter)	83.2 (40.5–90.3)	286 (90.3–987.2)***
% Macrophages	92.8 (73.6–96.6)	95.8 (91.4–98.6)***
% Lymphocytes	5.8 (2.4–25)	2.5 (1–7.8)**
% Neutrophils	1.4 (0.2–6.4)	0.6 (0.2–2.8)
% Eosinophils	0.4 (0–6)	0.1 (0–2)

Data presented as median (min-max). Abbreviations: FVC, forced vital capacity; FEV1, forced expiratory volume in one second.

In the group of smokers there was one ex-smoker, who quit smoking only recently (two months) before bronchoscopy,

*(*p*<0.05),

**(*p*<0.01) and,

***(p<0.001).

### Sample preparation

After local anaesthesia, a fibreoptic bronchoscope was wedged in one of the sub-segments of the middle lobe and five 50 mL aliquots of 37°C and sterile phosphate buffered saline (PBS) were instilled and aspirated. The BAL fluid was transferred to the new tubes after centrifugation and the cell pellet was resuspended in RPMI 1640 (Sigma-Aldrich). The BAL cell differential counts were determined by staining cytospin slides with May–Grünwald–Giemsa. Peripheral blood samples were obtained by venipuncture and peripheral blood mononuclear cells (PBMCs) were separated from heparinized peripheral blood by Ficoll-Hypaque (Amersham Pharmacia Biotech) gradient centrifugation. Three million BAL cells and three million PBMC were re-suspended in RPMI 1640 supplemented with HEPES (2 mM), human serum (Sigma-Aldrich) (10%), penicillin-streptomycin (1%) and L-glutamine (1%).

### Intracellular cytokine staining and flow cytometry

Three million BAL cells and three million PBMC were transferred to separated tubes for intracellular staining and flow cytometry.

The T cell cytokines IFNγ, IL-17 (also known as IL-17A), IL-10 and TNFα plus the T cell transcription factors T-bet, Foxp3 and Helios were selected for intracellular staining. After harvest, BAL cells and PBMC were stimulated *ex vivo* with anti-CD3 (clone UCHT1, mIgG1, Biolegend, San Diego, CA, USA) together with anti-CD28 (clone CD28.2, mIgG1, BD, Mountain View, CA, USA) in 37°C, humidified atmosphere of 5% CO_2_ in air. Unstimulated BAL and PBMC cells in medium alone were used as negative controls. Protein transport inhibitor (containing Brefeldin A) (BD, Mountain View, CA, USA) was added to each tube after 4 h incubation followed by 12 h incubation for a total of 16 h incubation. Extracellular staining by monoclonal anti-CD3-FITC (clone UCHT1, mIgG1, Dako, Denmark), anti-CD4-APC-Cy7 (clone OKT4, mIgG1, Biolegend, USA) and anti-CD8-PerCP-Cy5.5 (clone SK1, mIgG1, BD, USA) was performed on all samples. The intracellular staining was performed on BAL and PBMC samples following fixation and permeabilization (all buffers and reagents from eBioscience, USA). Both BAL cells and PBMC were stained in two separate antibody panels (S1 Table) by monoclonal anti-IFNγ-PE-Cy7 (clone B27, mIgG1, BD, USA), anti-IL-17-PE (clone SCPL1362, mIgG1, BD, USA), anti-TNFα-V450 (clone MAb11, mIgG1, BD, USA), anti-IL-10-APC (clone JES3-19F1, Rat IgG2, Biolegend, USA), anti-T-bet-FITC (clone 4B10, mIgG1, Biolegend, USA), anti-FOXP3-Pacific Blue (clone 206D, mIgG1, Biolegend, USA), and anti-Helios-PE (clone 22F6, mHamster IgG, Biolegend, USA). Matched isotype controls were used for each cytokine and transcription factor.

All samples were stained with aqua fluorescent reactive dye (Live/Dead fixable dead cell stain kits, Invitrogen, USA) and all gating was done on live cell populations. Flow cytometric analysis was performed on a BD FACS Canto II flow cytometer (BD Biosciences, USA), and the data were processed using the FACSDiva software (BD Biosciences, USA). All cytokine data following stimulation are reported after background deduction, see above.

### Statistical analysis

The statistical analysis of the data was performed utilizing GraphPad PRISM 4.03 (GraphPad Software Inc., San Diego, CA, USA). The Mann–Whitney U test and Wilcoxon signed rank test was used for comparisons between independent and dependent parameters, respectively. Correlation analysis was performed by Spearman’s rank correlation test and analysis of data for more than two groups were determined by one-way ANOVA test and followed by Dunn’s or Tukey’s post test for non-parametric or parametric values respectively. A *p*-value < 0.05 was considered to define statistical significance.

## Results

### Subject characteristics

**Fig 1:**
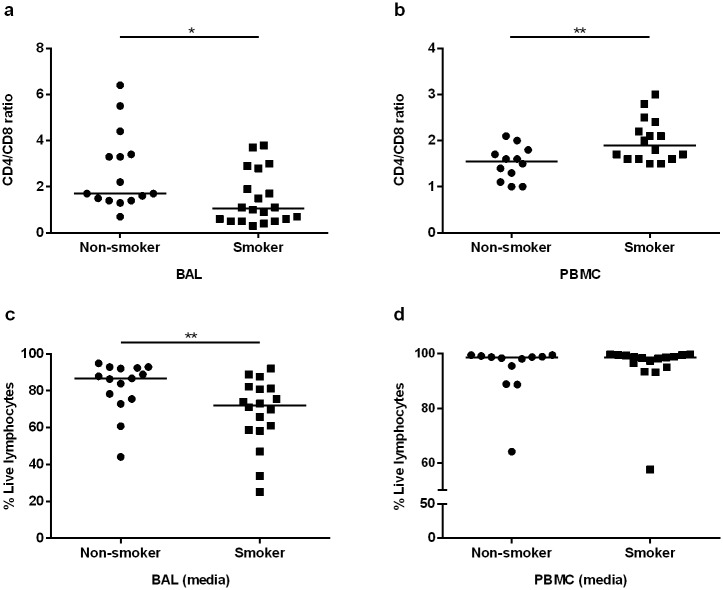
CD4/CD8 ratio and percentage of live lymphocytes in BAL and blood from healthy smokers and non-smokers (in unstimulated sample). Cells were stained and characterized by flow cytometry to characterize CD4/CD8 ratio. Aqua fluorescent reactive dye (Live/Dead fixable dead cell stain kits) was used for all samples in order to gate on live cells, *(*p*<0.05) and **(*p*<0.01).

We obtained BAL cell and PBMC samples from eighteen smokers and fifteen never-smokers. The basic characteristics are listed in Table 1, demonstrating that our two study groups were well-matched with reference to ascertaining normal lung function (assessed as ventilatory capacity). The percentages of macrophages in BAL were clearly increased and the corresponding percentage of lymphocytes were clearly decreased in smokers in comparison with never-smokers. However, there were no reproducible differences in the percentages of BAL neutrophils, eosinophils or basophils. Notably, there was a markedly lower CD4/CD8 ratio of BAL T cells in smokers than in never-smokers (Fig 1a) while this ratio displayed the opposite pattern in blood (Fig 1b). All of our findings were based on gating of live cells and we detected a significantly increased proportion of dead BAL lymphocytes in samples from healthy smokers after 16 hours incubation while no such difference between groups was seen in peripheral blood lymphocytes (Fig 1c and 1d).

### Cytokine production and transcription factors in BAL and PBMC of healthy smokers and never-smokers

**Fig 2:**
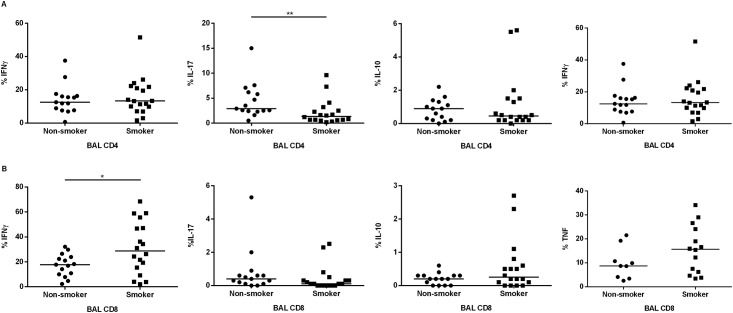
Percentage of IFNγ, IL-17, IL-10 and TNF producing cells in BAL CD4+ and CD8+ T cells of healthy smokers and never-smokers. T cells were stimulated with anti-CD3/CD28 and following intracellular cytokine staining the frequency of cells expressing each cytokine was determined by flow cytometry. The statistical analyses were done after background deduction *(*p*<0.05) and **(*p*<0.01).

The capacity for IFNγ, IL-17, IL-10 and TNFα production in BAL and PBMC by CD4+ and CD8+ T cells in response to *ex vivo* stimulation by anti-CD3/CD28 was examined utilizing flow cytometry. In all analyses, the cytokine content of cells in the unstimulated control sample was deducted from that of the stimulated cells, so the results reflect the response to *in vitro* stimulation. We found that smokers displayed lower frequencies of IL-17-producing BAL CD4+ T cells compared with never-smokers (*p*<0.01) (Fig 2a). There was a similar trend for TNFα-producing BAL cells in smokers, whereas there is no statistically significant difference for other cytokines between smokers and never-smokers (Fig 2a).

The pattern of cytokine-containing BAL CD8+ T cells indicated a significantly higher frequency of IFNγ-producing CD8+ T cells in BAL compared to the never-smokers (*p*<0.05), and we found no statistically significant difference between smokers and never-smokers in case of other cytokines (Fig 2b).

No differences were detected between smokers and never-smokers for any of cytokines in PBMC (S1 Fig). There was a decreased IL-17+/IL-10+ ratio for BAL CD4+ T cells in smokers compared with never-smokers (p<0.01) (S2 Fig).

**Fig 3:**
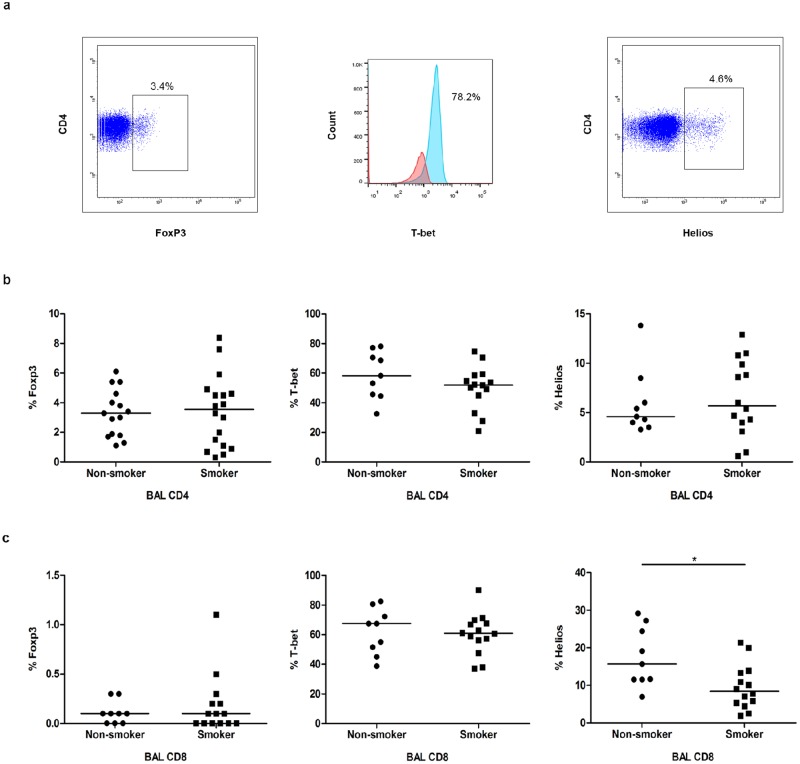
Transcription factors analysis. (a) Representative flow cytometry plots of BAL CD4+ T cells to detect Foxp3, T-bet and Helios. Foxp3+ and Helios+ T cells constitute distinct populations, while for gating on T-bet+ cells we set it according to isotype control and also matching with IFNγ producing cells (S3 Fig). The percentages of cells expressing each transcription factor was compared between smokers and non-smokers in (b) BAL CD4+ and (c) BAL CD8+ T cells, *(*p*<0.05).

There was no clear difference between smokers and never-smokers regarding T-bet, Foxp3 and Helios expression in BAL or blood CD4+ and CD8+ T cells with one exception: There was a lower frequency of Helios positive cells in BAL CD8+ T cells of smokers (p<0.05) (Fig 3a, 3b and 3c, S3 Fig and data not shown). The frequencies of CD4+ T cells expressing Foxp3+ and T-bet+ were higher in BAL compared to blood in both smokers and never-smokers (S4 Fig).

### Distinctive regulatory T cells in smokers

**Fig 4:**
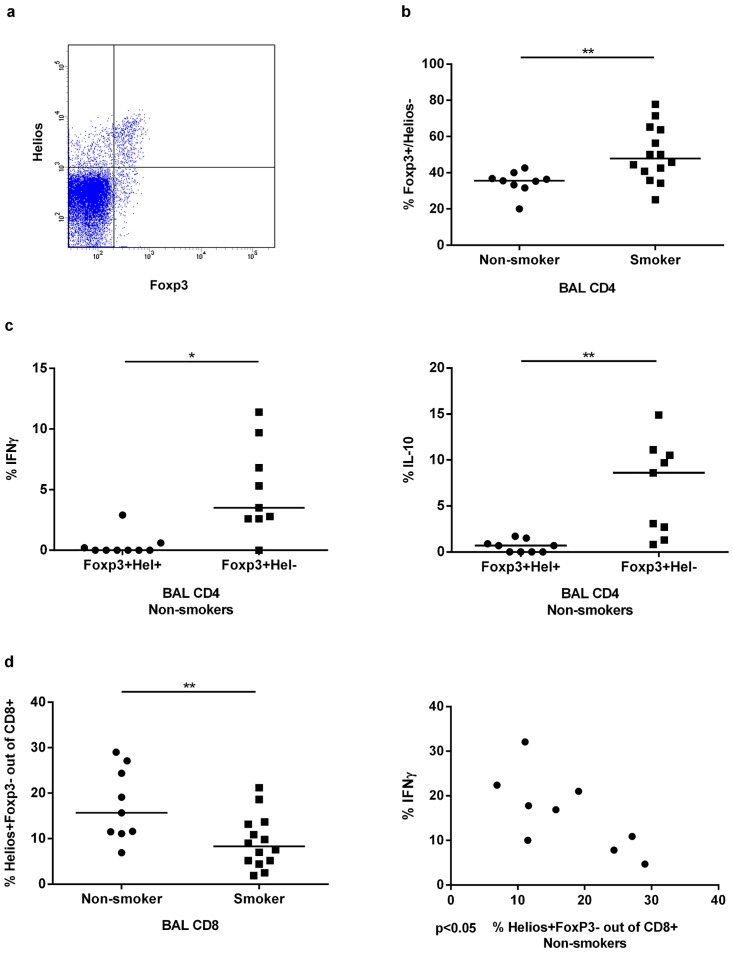
Analysis of regulatory T cells. (a) Representative flow cytometry plot to detect the combination of Foxp3+ and/or Helios+ BAL CD4+ T cells. (b) The fraction of BAL CD4+ Foxp3+ T cells that are Helios- compared in smokers and non-smokers. (c) Comparison of IFNγ and IL-10 production between Foxp3+Helios+ and Foxp3+Helios- BAL CD4+ T cells in non-smokers. (d) Comparison of the percentages of Foxp3- Helios+ BAL CD8+ T cells between smokers and non-smokers and correlation of this population with BAL CD8+ IFNγ producing cells in non-smokers, *(*p*<0.05) and **(*p*<0.01).

Because the expression of Helios may discriminate the origin and function of Treg cells, we compared the fractions of Helios+ and Helios- Foxp3+ regulatory T cells between smokers and never-smokers (Fig 4a). We then found a clearly higher fraction of Helios- Tregs in smokers compared with never-smokers (p<0.01) (Fig 4b).

In addition, we analyzed the cytokine content (IFNγ and IL-10) of Helios+ and Helios- regulatory Foxp3+ T cells. Our findings indicated that for both cytokines, their content in Tregs of never-smokers was mainly confined to the Helios- subset (the low proportion of Tregs to total BAL cells in smokers precluded a similar analysis in these subjects). In never-smokers, there was a significantly larger proportion of Helios- Tregs that expressed IFNγ or IL-10 compared to Helios+ Tregs (p<0.05) and (p<0.01) respectively (Fig 4c).

We also performed an analysis of postulated CD8+ regulatory T cells, since there have been recent claims that Helios+Foxp3- CD8+ T cells possess a regulatory function. We found a reproducible reduction of this subset in smokers compared to non-smokers (p<0.01) (Fig 4d). Moreover we found a statistically significant negative correlation between the fractions of Helios+Foxp3- cells and IFNγ producing cells in the BAL CD8+ T cell compartment of never-smokers (p<0.05). In contrast, no such correlation was detected in BAL CD8+ of smokers.

### Cytokine production by Foxp3^-^/Helios^+^ and Foxp3^-^/Helios^-^ effector T cells

**Fig 5:**
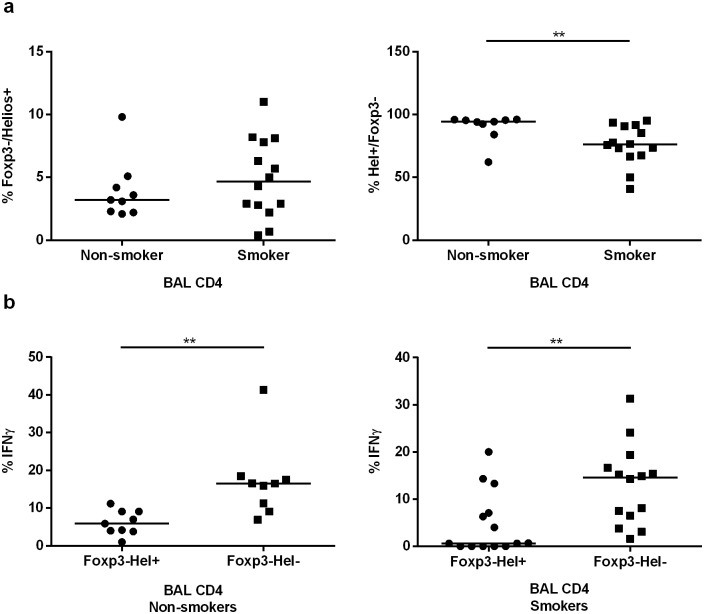
Cytokine production by Foxp3- effector T cells. (a) The fraction of BAL CD4+ Foxp3- T cells that are Helios+ compared in smokers and non-smokers; and the fraction of BAL CD4+ Helios+ T cells that are Foxp3- compared in smokers and non-smokers (b) Comparison of IFNγ production between Foxp3-Helios+ and Foxp3-Helios- BAL CD4+ T cells in non-smokers and smokers.

The fraction of BAL Foxp3- effector T cells which were Helios+ or Helios- were compared for smokers and never-smokers, but there was no difference between these groups in this respect. However, there was a lower frequency of Helios+ T cells which were Foxp3- in smokers (Fig 5a).

The cytokine contents of Helios+ and Helios- effector Foxp3- T cells were characterized in smokers and never-smokers. We found that the proportions of cells producing IFNγ after stimulation was significantly higher in the Foxp3-/Helios- BAL CD4+ T cell subset compared to the Foxp3-/Helios+ subset, in smokers as well as never-smokers (p<0.01) (Fig 5b). No corresponding differences between the effector cell subsets were detected in the case of IL-10 expression (data not shown).

### Production of cytokines by BAL T cells versus history of daily cigarette consumption

**Fig 6:**
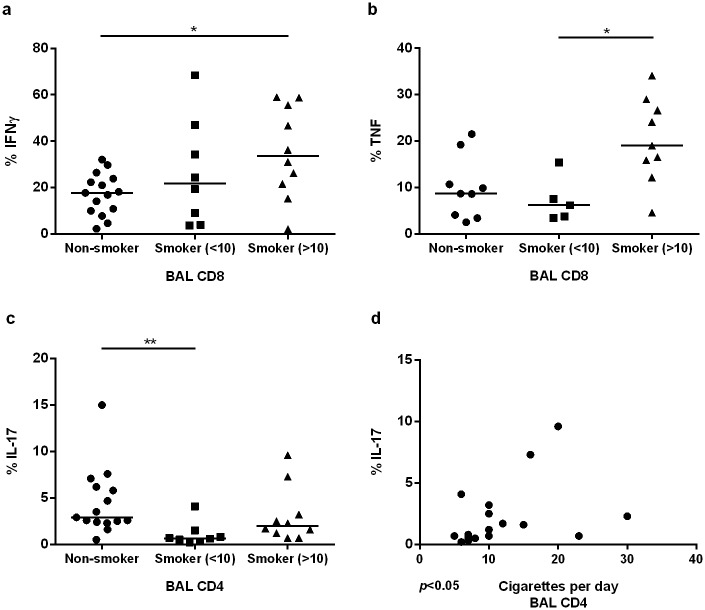
Cytokine production in relation to daily cigarette consumption. (a) Comparison of percentages of BAL CD8+ IFNγ producing cells, (b) BAL CD8+ TNF producing cells and (c) BAL CD4+ IL-17 producing cells between non-smokers, smokers with less than 10 cigarettes per day and smokers with equal to or higher than 10 cigarettes per day. One way Anova was done for statistics analysis between groups. (d) Correlation between percentage of BAL CD4+ IL-17 producing cells and number of cigarettes per day in healthy smokers, (r = 0.49 *(*p*<0.05) and **(*p*<0.01).

To determine the impact of ongoing, daily cigarette consumption on the cytokine production in BAL and blood T cells, we divided the healthy smokers into smokers with less or equal to/more than 10 cigarettes per day (named moderate and heavy smokers respectively). Heavy smokers had a markedly higher frequency of BAL CD8+ cells, producing IFNγ compared to never-smokers (p<0.05) (Fig 6a) and of BAL CD8+ cells producing TNFα, compared to moderate smokers (p<0.05) (Fig 6b). In contrast, there was a clearly lower frequency of BAL CD4+ cells producing IL-17 among moderate smokers, compared with never-smokers (p<0.01). However, heavy smokers did not differ from never-smokers with regard to the production of IL-17 in a reproducible manner (Fig 6c). Furthermore, there was a positive correlation between the frequency of BAL CD4+ cells producing IL-17 and the history of daily cigarette consumption (p<0.05) (Fig 6d).

## Discussion

In the current study, we characterized the content of cytokines and transcription factors in lung and blood T cell subsets from clinically healthy and relatively young smokers with normal lung function in comparison with matching never-smoking controls. We found a reduced content of IL-17 in CD4+ BAL T cells and an increased content of IFNγ in CD8+ BAL cells. We also found an increased frequency of Foxp3+ Helios- Tregs in the smokers. We propose that the demonstrated alterations in Th17 and Tregs cells predispose for increased susceptibility to airway infection, the development of chronic inflammation and, possibly, autoimmune disorders.

The reduced CD4/CD8 ratio in BAL from the clinically healthy smokers with normal lung function, and corresponding increased ratio in peripheral blood, may reflect a preferential recruitment of CD8+ T cells to the airways, although other explanations also have been proposed [14, 15]. The reduced survival of BAL cells of smokers after *in vitro* culture may reflect *in vivo* exposure to cigarette smoke components known to induce both apoptosis and necrosis of lymphocytes [16].

There was an increased frequency of IFNγ-producing BAL CD8+ T cells in the clinically healthy smokers with normal lung function and this difference was mainly observed in the individuals with high daily tobacco load. It is known that in COPD, frequently caused by tobacco smoking, in particular lung CD8+ T cells display up-regulation of inflammatory mediators and cytotoxic molecules [17, 18]. Indeed, IFNγ is currently perceived as a key mediator in the development of lung tissue destruction in COPD by induction of different mediators and apoptosis [19]. Even though it has remained unclear to what extent previously demonstrated alterations in IL-17-producing cells has been due to tobacco smoking or COPD, their involvement in airway inflammation caused by tobacco smoke has been indicated before [2, 6]. Thus, the increased expression of the Th17-related cytokines and the potential role of IL-17 and IL-17 producing cells in driving the small airway inflammation in smokers has been suggested before [20–22] and an increased number of IL-17 producing cells in bronchial tissue from COPD patients compared with non-smokers has been reported [21, 23–25]. In contrast, our data on clinically healthy and relatively young smokers with normal lung function shows a reduced in intracellular content of IL-17 protein by *in vitro* stimulated BAL CD4+ T cells harvested from the sub-group of moderate smokers (defined as smoking less than 10 cigarettes per day) but a “normalized” IL-17 production in the sub-group of heavy smokers (smoking 10 or more cigarettes per day). In this context, we think that it is important to note that most previous studies have addressed the effect of smoking on T cells by investigating never-smokers and smokers who were matched with elderly COPD patients with reference to history of tobacco smoking (i.e. tobacco load), while, again, our present study was conducted by investigating clinically healthy and relatively young smokers with a completely normal lung function. Hypothetically, our data showing a decline in the content of IL-17 in BAL CD4+ T cells from smokers illustrate a feasible mechanistic explanation for bacterial colonization and increased susceptibility to infection in the lungs of smokers [26]. Interestingly, it was recently shown that in smokers with obstructive pulmonary disease including chronic bronchitis, lower systemic levels of IL-17 was associated with colonization by opportunistic pathogens in the airways [27]. The negative effect of smoking on adaptive immune responses in animal studies has been reported before [28]. At present, however, very little is known with reference to how systemic and local levels of IL-17 relate to one another in smokers.

Our findings of a marked increase in the proportion of Helios- Foxp3+ Tregs in BAL of clinically healthy smokers with normal lung function are of interest, since these immune cells have distinct functional characteristics. Specifically, we found that expression of IL-10 and IFNγ in Foxp3+ Tregs was almost exclusively confined to the Helios- subset in agreement with previous studies on human [29]. Obviously, if Helios- Tregs are potent suppressors, then the observed decrease in Th17 cells and other inflammatory cytokine producing cells in smokers may be ascribed to the higher frequency of these Helios- Tregs in smokers. Such a scenario would be in agreement with the previously published finding of Helios- Tregs having a higher suppressive capacity compared to Helios+ Tregs [29]. To us, it seems feasible that Helios- Tregs may respond to cigarette smoke by reducing their suppressive activity. The suppressive function of Foxp3+ regulatory T cells is enhanced by Helios induction in a mouse model [30]. However, the function of Helios in regulatory T cells and differences in suppressive function between Foxp3+Helios+ and Foxp3+Helios- regulatory T cells needs to be further investigated. Moreover, it should be stressed that Helios is not expressed just by regulatory T cells and expression of substantial levels of Helios by effector T cells in response to (auto-)antigen stimulation has been reported by Ross *et al* [12]. They demonstrated, in a TCR transgenic mouse model, that Helios+ effector T cells are anergic T cells with less proliferative activity, less IL-2 production and poor survival and proposed that Helios might have a role in T cell tolerance. In agreement with this demonstration, we found that Helios+ CD4+ T effector cells produced less IFNγ compared to Helios- effector cells (both in smokers and non-smokers). In addition, our findings indicate a tendency to downregulation of cytokine expression by the minor subset of Helios+ effector cells in smokers compared to non-smokers.

In the clinically healthy young smokers, virtually all CD8+ cells were Foxp3- and there was a reduction in the proportion of Helios+ Foxp3- cells in BAL CD8+ cells, a reduction that may relate to T cell regulation. Helios is essential for CD8+ Treg development [31] and CD8+ regulatory T cells depend on Helios to maintain their suppressive cytolytic activity [32]. If Helios+ CD8+ T cells, or a subset thereof, are indeed Treg cells, whose numbers are reduced by smoking, this may affect immune regulation in the lung. Indeed, our results revealed a negative correlation between Helios+ Foxp3- CD8+ cells and CD8+ IFNγ producing cells in the clinically healthy never-smokers, while there was no such correlation in BAL of smokers. Since we observed an enhanced IFNγ production by CD8+ cells in BAL of smokers, it can be speculated that this phenomenon was caused by a defect in CD8+ regulatory T cells.

We found that an increased daily quantity of cigarettes was associated with higher frequencies of BAL CD8+ T cells producing the pro-inflammatory cytokines IFNγ and TNFα, while a low daily cigarette consumption was associated with a reduction of IL-17 producing BAL CD4+ T cells. Importantly, this highlights the importance of taking daily cigarette consumption into account when comparing results of different studies. The finding that the dynamics of cytokine production by airway T cells in relation to smoke exposure appears to depend both on T cell subset and what cytokine is studied merits further investigation.

Associations of smoking with autoimmune disorders and infections have been shown in a number of studies. There is strong evidence indicating that the development of many autoimmune diseases such as rheumatoid arthritis (RA), systemic lupus erythematous, Crohn's disease and multiple sclerosis (MS) are linked to smoking [1]. MS and RA are examples of autoimmune diseases where cigarette smoking strongly increases the risk of developing disease in people with a particular genetic susceptibility [33, 34]. Similarly, smoking is a risk factor for myositis with anti-Jo-1 antibodies in a subset of patients [35]. The association of these autoimmune disorders with certain HLA molecules can be explained by the presence of particular antigens derived from altered self-proteins which are involved in the initiation of the inflammatory responses. Smoking also has a strong association with induction of infections [26]. Although the exact mechanisms behind inducing autoimmunity remain to be clarified there is evidence to support the role of infection in triggering autoimmunity [36]. Activation of self-reactive T cells and B cells following infections can be explained by different mechanisms such as molecular mimicry (cross-recognition between a self and infectious agent by the same lymphocytes) and bystander activation (activation of immune cells against infectious agents results also in the activation self-reactive lymphocytes) [37, 38].

In conclusion, our study indicates that even relatively young tobacco smokers with a normal lung function display a distinctly altered immune status locally in the lungs. These smokers have increased IFNγ production by CD8+ T cells, a decreased frequency of Th17 cells, and a change in the phenotype of the regulatory T cell population e.g. increased proportions of CD4+ Foxp3+ Helios- regulatory T cells and decreased proportions of CD8+ Helios+ Foxp3- (postulated regulatory) T cells in the lungs. As a result, there may be a functionally altered regulatory capacity in these cells. The observed alterations within these young smokers are likely to have consequences for the susceptibility of infections, which may also relate to the breaking of self-tolerance and the induction of autoimmunity, potentially leading to chronic inflammation.

## Supporting Information

S1 Fig Paired analysis of cytokine producing cells in BAL and PBMC of CD4+ and CD8+ T cells from healthy smokers and never-smokers. (TIF)

S2 Fig The balance between BAL CD4+ IL-17 producing T cells and regulatory T cells markers. (TIF)

S3 Fig T-bet expression by BAL CD4 T-cells. (TIF)

S4 Fig Paired analysis of transcription factors between BAL and blood CD4+ T cells from healthy smokers and non-smokers. (TIF)

S1 Table Monoclonal antibodies and panels used for staining of T-cells. (DOCX)
