# Supplementary figures and images for: Distinctive Regulatory T Cells and Altered Cytokine Profile Locally in the Airways of Young Smokers with Normal Lung Function
Source: PLoS One. 2016 Oct 31;11(10):e0164751. doi: 10.1371/journal.pone.0164751 (PMC5087844; doi:10.1371/journal.pone.0164751)

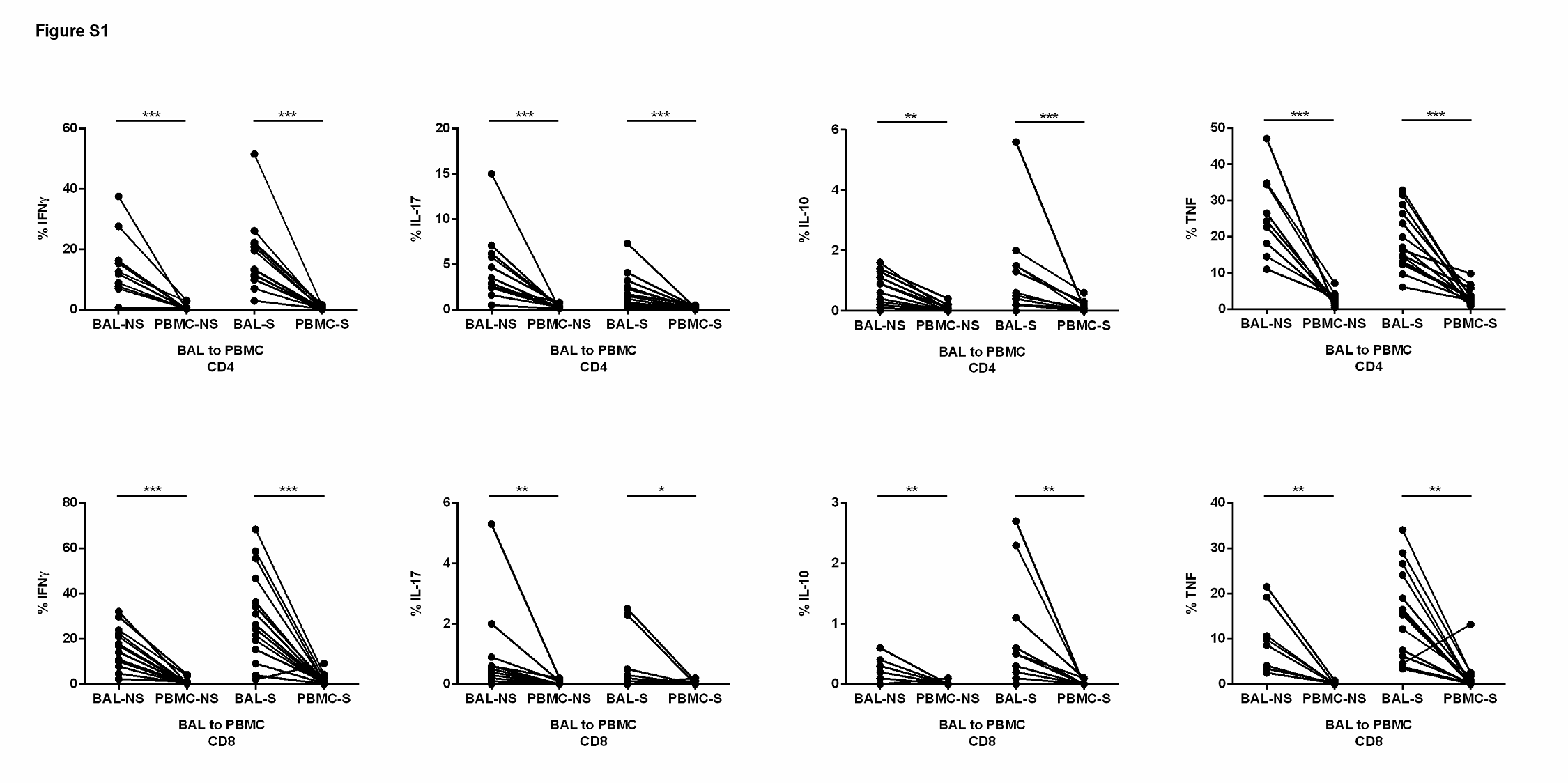

Supplement: S1 Fig — (TIF) [file pone.0164751.s001.tif]

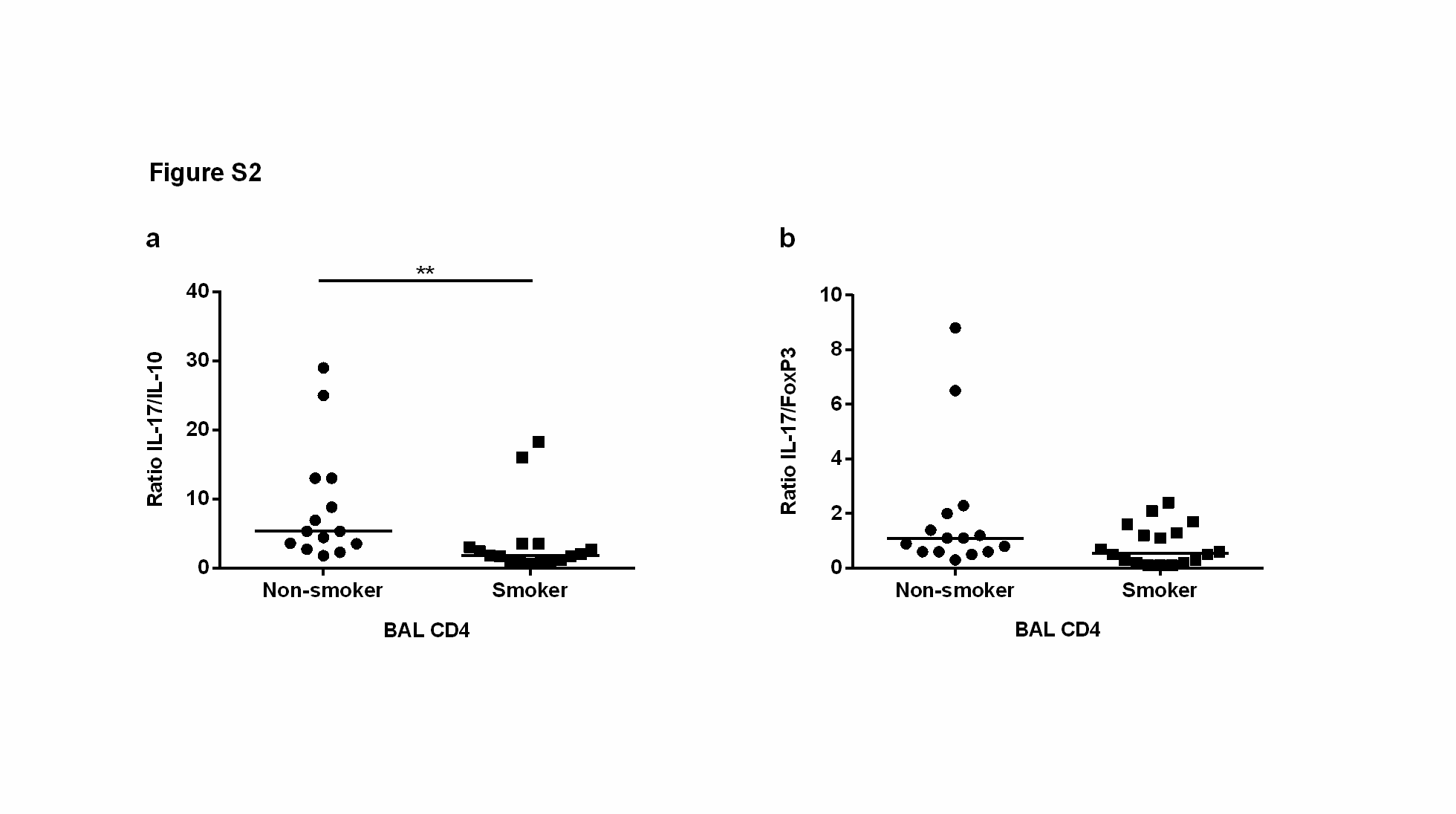

Supplement: S2 Fig — (TIF) [file pone.0164751.s002.tif]

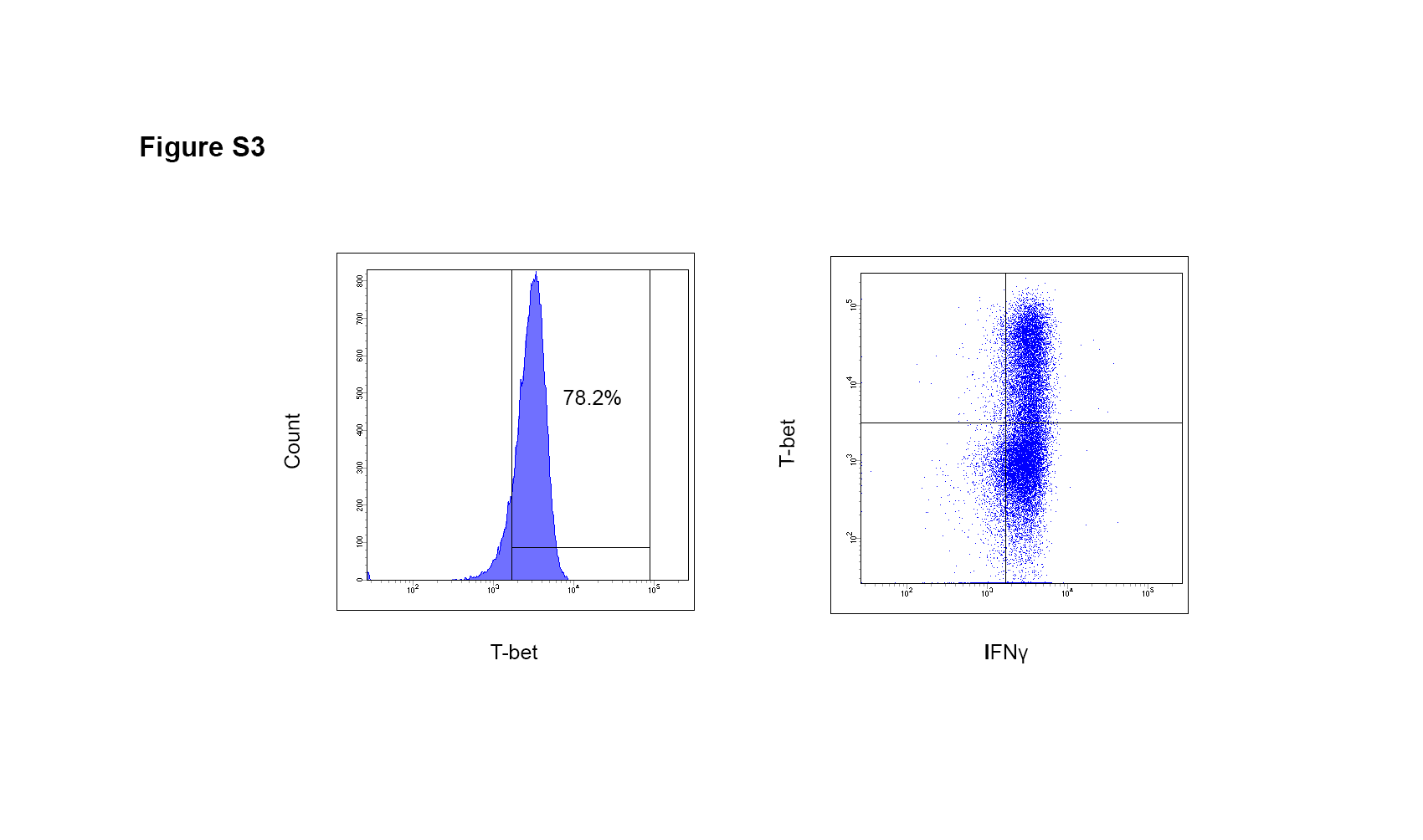

Supplement: S3 Fig — (TIF) [file pone.0164751.s003.tif]

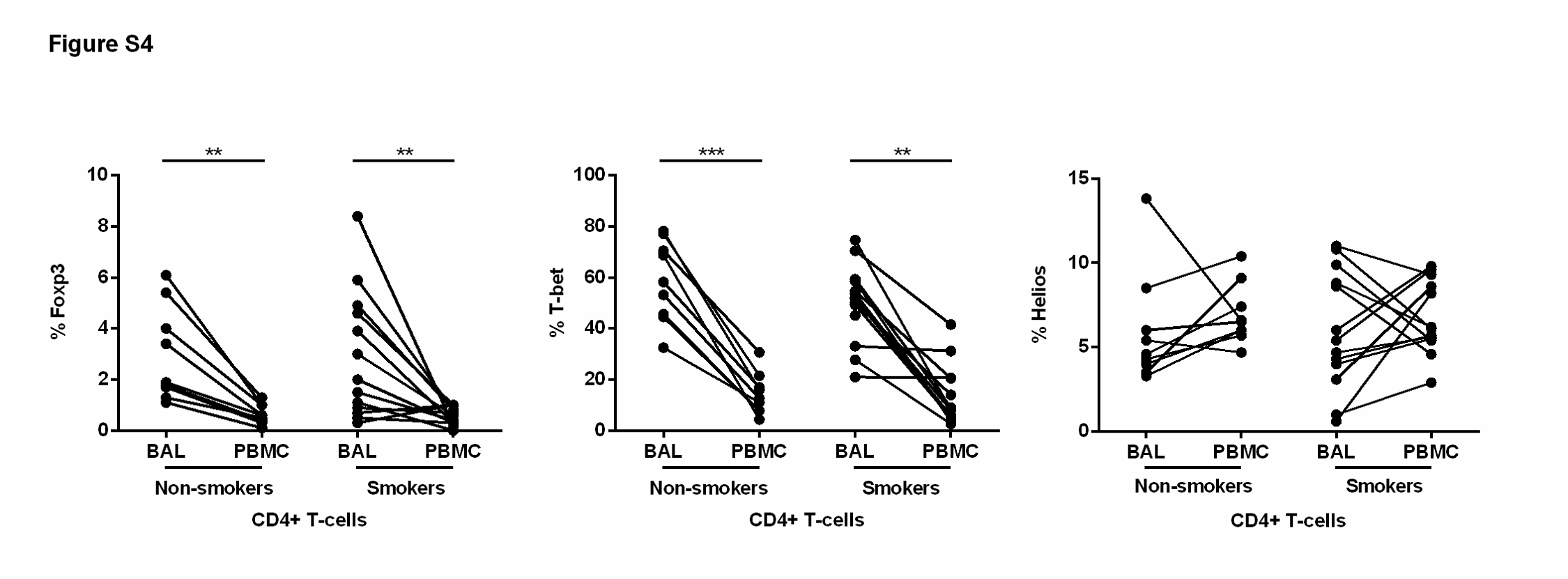

Supplement: S4 Fig — (TIF) [file pone.0164751.s004.tif]
