# Supplementary material for: Distinctive Regulatory T Cells and Altered Cytokine Profile Locally in the Airways of Young Smokers with Normal Lung Function
Source: PLoS One. 2016 Oct 31;11(10):e0164751. doi: 10.1371/journal.pone.0164751 (PMC5087844; doi:10.1371/journal.pone.0164751)
Supplement: S1 Table — (DOCX) [file pone.0164751.s005.docx]

**Supporting information**

**Distinctive regulatory T cells and altered cytokine profile locally in the airways of young smokers with normal lung function**

**S1 Table: Monoclonal antibodies and panels used for staining of T-cells.**

BAL and blood cells were stained with two separate antibody panels. All samples were stained with aqua fluorescent reactive dye (Live/Dead fixable dead cell stain kits, Invitrogen, USA) in both panels in order to exclude dead cells from the analysis. For each cytokine and transcription factor, cells were separately stained with matched isotype controls.

Panel I

| **Antibody Specificity** | **Fluorochrome** | **Clone** | **isotype** | **Company** |
| --- | --- | --- | --- | --- |
| CD3 | FITC | UCHT1 | mIgG1 | Dako, Denmark |
| CD4 | APC-Cy7 | OKT4 | mIgG1 | Biolegend, USA |
| CD8 | PerCP-Cy5.5 | SK1 | mIgG1 | BD, USA |
| IFNγ | PE-Cy7 | B27 | mIgG1 | BD, USA |
| IL-17 | PE | SCPL1362 | mIgG1 | BD, USA |
| TNF | V450 | MAb11 | mIgG1 | BD, USA |
| IL-10 | APC | JES3-19F1 | Rat IgG2 | Biolegend, USA |

Panel II

| **Antibody Specificity** | **Fluorochrome** | **Clone** | **isotype** | **Company** |
| --- | --- | --- | --- | --- |
| CD4 | APC-Cy7 | OKT4 | mIgG1 | Biolegend, USA |
| CD8 | PerCP-Cy5.5 | SK1 | mIgG1 | BD, USA |
| IFNγ | PE-Cy7 | B27 | mIgG1 | BD, USA |
| IL-10 | APC | JES3-19F1 | Rat IgG2 | Biolegend, USA |
| T-bet | FITC | 4B10 | mIgG1 | Biolegend, USA |
| Foxp3 | Pacific Blue | 206D | mIgG1 | Biolegend, USA |
| Helios | PE | 22F6 | Hamster IgG | Biolegend, USA |

**S1 Fig: Paired analysis of cytokine producing cells in BAL and PBMC of CD4+ and CD8+ T cells from healthy smokers and never-smokers.**

Paired analysis of cytokine producing cells in response to anti CD3/CD28 stimulation compared between BAL and blood samples of smokers (S) and non-smokers (NS), *(*p*<0.05), **(*p*<0.01) and ***(p<0.001).

**S2 Fig: The balance between BAL CD4+ IL-17 producing T cells and regulatory T cells markers.**

(A) Ratio of IL-17/IL-10 positive cells and (B) IL-17/Foxp3 positive cells in the BAL CD4+ T cell compartment in smokers and non-smokers. Since smoking was associated with changes both in Th17 cells and Treg cells, and the balance of these subsets is believed to be of importance for inflammation-induced pathology (1, 2), we compared the ratios of IL-17 to regulatory markers in smokers and never-smokers. Indeed, there was a decreased IL-17^+^/IL-10^+^ ratio for BAL CD4+ T cells in smokers compared with never-smokers (p<0.01) (S2A Fig). Moreover, there was a trend towards a decreased IL-17/Foxp3 ratio for BAL CD4+ T cells in smokers compared to never-smokers, a trend that did not prove statistically significant (p=0.08), however (S2B Fig). No corresponding differences were observed for the balance between Th1 and regulatory T cells among smokers and never-smokers (data not shown). Our finding of a reduced ratio of IL-17- to IL-10-producing CD4+ BAL T cells in clinically healthy and young smokers is seemingly contradictive to the findings in a recent study on an animal model of smoke-exposure (3). In that particular animal study, however, the investigators utilized a very high dose of smoke exposure to induce COPD-like pathology.

**S3 Fig: T-bet expression by BAL CD4 T-cells.**

Representative flow cytometry plots of BAL CD4 T-cells to detect T-bet expression. T-bet+ CD4+ T cells did not constitute a distinctive population. In order to gate on them we set the cut off according to the isotype control and also matched it with a distinctly T-bet+ fraction of IFNγ producing cells.

**S4 Fig: Paired analysis of transcription factors between BAL and blood CD4+ T cells from healthy smokers and non-smokers.**

Paired analysis of transcription factors (FoxP3, T-bet and Helios) in CD4+ T cells compared between BAL and blood samples of smokers and non-smokers, **(*p*<0.01) and ***(p<0.001). The content of all three transcription factors (MFI values) in transcription factor expressing BAL CD4+ and CD8+ T cells was very similar for smokers and never-smokers (data not shown). Comparison of transcription factor-positive cell frequencies between BAL and blood showed that Foxp3 and T-bet were expressed by higher frequencies of CD4+ T cells among BAL cells compared with PBMC.

**References:**

1. Niu Q, Cai B, Huang ZC, Shi YY, Wang LL. Disturbed Th17/Treg balance in patients with rheumatoid arthritis. Rheumatol Int. 2012;32(9):2731-6.

2. Zhao J, Lloyd CM, Noble A. Th17 responses in chronic allergic airway inflammation abrogate regulatory T-cell-mediated tolerance and contribute to airway remodeling. Mucosal Immunol. 2013;6(2):335-46.

3. Wang H, Peng W, Weng Y, Ying H, Li H, Xia D, et al. Imbalance of Th17/Treg cells in mice with chronic cigarette smoke exposure. Int Immunopharmacol. 2012;14(4):504-12.
